# Supplementary material for: Therapeutic Drug Monitoring in Children and Adolescents: Findings on Fluoxetine from the TDM-VIGIL Trial
Source: Pharmaceutics. 2023 Aug 25;15(9):2202. doi: 10.3390/pharmaceutics15092202 (PMC10534581; doi:10.3390/pharmaceutics15092202)
Supplement: Supplementary file 1 [file pharmaceutics-15-02202-s001.zip › Supplement S1 Rating scheme adherence.pdf]

Supplement S1

Assessment of adherence to medication:

The following input options were available to treating physicians to assess adherence and were stored in the database.

1- certain
2- probably
3- uncertain
4- demonstrable intake error
5- not yet known
