# Supplementary material for: Therapeutic Drug Monitoring in Children and Adolescents: Findings on Fluoxetine from the TDM-VIGIL Trial
Source: Pharmaceutics. 2023 Aug 25;15(9):2202. doi: 10.3390/pharmaceutics15092202 (PMC10534581; doi:10.3390/pharmaceutics15092202)
Supplement: Supplementary file 1 [file pharmaceutics-15-02202-s001.zip › Supplement S2 Methods TDM laboratory FLX and NORFLX 230818.pdf]

Supplement S2

Therapeutic Drug Monitoring of Fluoxetine and Norfluoxetine – Laboratory methods

Therapeutic drug monitoring (TDM) was performed according to the guidelines of the Working Group on Neuropsychopharmacology and Pharmacopsychiatry (AGNP). Blood collection from cubital veins in 7.5-ml monovettes without anticoagulants and additives took place at a steady-state trough level (after at least 14 days of consistent fluoxetine dosage; between 10 and 24 hours after the last dose) before the first daily drug intake. The blood was centrifuged at 1800 × *g* for 10 minutes and analyzed immediately (samples from Wuerzburg) or within a few days after postage to the TDM laboratory in Wuerzburg. Analysis of serum samples demonstrated that fluoxetine/norfluoxetine is not degraded under the following storage conditions: samples at +4°C in refrigerator for up to 1 week of storage and, for a longer period, at -20°C.

Fluoxetine serum concentrations were analyzed by an automated column-switching method coupled to an isocratic high-performance liquid chromatography system and a variable ultraviolet detector (Agilent 1200 Series; Agilent, Waldbronn, Germany). Chemicals and solvents of the highest level of purity and sertraline for calibration were purchased commercially from Sigma–Aldrich Chemie GmbH (Taufkirchen, Germany). A different washing and analytical eluent, different columns, and a different emission wavelength were used to achieve better separation of both substances from potential comedications.

The cycle of operation started with the injection of a 100-µl serum sample onto the extraction column (PerfectBond Vorsäulenkartusche 10×4, 0 mm CN 20 mm; MZ-Analysentechnik, Mainz, Germany) using a washing eluent of 10% (*v*/*v*) acetonitrile in deionized water at a flow rate of 1.25 ml/min. After 4 minutes, the electric six-port valve switched and the second pump transported the sample by back-flush mode onto the analytical column (MN-EC 150/4.6 NUCLEDUR 100-3 CN RP; Macherey–Nagel, Düren, Germany). The mobile phase contained 45% (*v*/*v*) acetonitrile and 10 mmol/l K_2HPO_4 in deionized water adjusted to pH 6.3 with H_3HPO_4. The flow rate was 1.25 ml/min at +30°C. The retention time was for fluoxetine 8.8 min and for norfluoxetine 8.1 min. The switching valve was reset after 14 minutes.

The emission wavelength of the ultraviolet detector was set at 210 nm. Data acquisition and integration were performed by means of the HP ChemStation (Agilent, Waldbronn, Germany). The absolute extraction recovery for fluoxetine was 93% and for norfluoxetine was 97%. The intra-assay coefficients of variation determined for both from 10 analyses (133.5 and 534 ng/ml) were in general less than 1%. The interassay variability for the analyte was in general less than 2.4%. The lower limit of quantification for both was 10.0 ng/ml. The method was linear in a range of 10–1335 ng/ml (R^2 = 0.99).
